# MDSCs enhance tumor cell proliferation in a caspase-1 related inflammasome cytokines dependent way

**DOI:** 10.1186/2051-1426-3-S2-P317

**Published:** 2015-11-04

**Authors:** Qi Zeng, Jesse Qualliotine, Richard Blosser, Drew Pardoll, Young Kim

**Affiliations:** 1 Johns Hopkins University, Baltimore, MD, USA; 2 Johns Hopkins University School of Medicine, Baltimore, MD, USA

## 

Myeloid-derived-suppression cells (MDSCs) are believed to be an important immune evasion mechanism by suppressing T cells. We investigated whether MDSCs have direct T cell independent pro-carcinogenic effect on tumor cells. We sorted the monocytic CD14^+^/CD11b^+^/HLA-DR^low^ MDSCs from head and neck squamous cell carcinoma (HNSCC) patients and found that MDSCs increased the proliferation index of HNSCC cells. Similar results were seen from co-culturing murine MDSCs and CT26 and B16 cells. Supernatant from the MDSCs were found to secrete inflammasome cytokines IL-1b and IL-18. MDSC's enhancement of proliferative index of the tumor was found to be caspase-1 dependent using FLICA. To test this in vivo, T cell depleted caspase-1 null mice showed significant decrease in tumor growth rate. To confirm the importance of myeloid inflammasome signaling in carcinogenesis, we suppressed MyD88 gene in tumor cell line Cal27 and found that the ability of MDSCs to promoting tumor proliferation is diminished. Taken together, our findings demonstrate that MDSCs can promote tumor cells proliferation on a inflammasome cytokines dependent manner.

